# Human Papillomavirus 16 Oncoproteins Downregulate the Expression of miR-148a-3p, miR-190a-5p, and miR-199b-5p in Cervical Cancer

**DOI:** 10.1155/2018/1942867

**Published:** 2018-11-29

**Authors:** Mi-Soon Han, Jae Myun Lee, Soo-Nyung Kim, Jae-Hoon Kim, Hyon-Suk Kim

**Affiliations:** ^1^Department of Medicine, Graduate School of Yonsei University, 50-1 Yonsei-ro, Seodaemun-gu, Seoul 03722, Republic of Korea; ^2^Department of Laboratory Medicine, U2 Clinical Laboratories, Jangwon Medical Foundation, 68 Geoma-ro, Songpa-gu, Seoul 05755, Republic of Korea; ^3^Department of Microbiology and Immunology, Yonsei University College of Medicine, 50-1 Yonsei-ro, Seodaemun-gu, Seoul 03722, Republic of Korea; ^4^Department of Obstetrics and Gynecology, Konkuk University Medical Center, 120-1 Neungdong-ro, Gwangjin-gu, Seoul 05030, Republic of Korea; ^5^Department of Obstetrics and Gynecology, Gangnam Severance Hospital, Yonsei University College of Medicine, 211 Eonju-ro, Gangnam-gu, Seoul 06273, Republic of Korea; ^6^Department of Laboratory Medicine, Severance Hospital, Yonsei University College of Medicine, 50-1 Yonsei-ro, Seodaemun-gu, Seoul 03722, Republic of Korea

## Abstract

Almost all cervical cancers are associated with human papillomavirus (HPV); however, the majority of women infected with this virus do not develop cervical cancer. Therefore, new markers are needed for reliable screening of cervical cancer, especially in relation to HPV infection. We aimed to identify potential microRNAs that may serve as diagnostic markers for cervical cancer development in high-risk HPV-positive patients. We evaluated the microRNA expression profiles in 12 cervical tissues using the hybridization method and verified them by quantitative polymerase chain reaction (qPCR). Finally, we evaluated the effects of HPV16 oncoproteins on the expression of selected microRNAs using cervical cancer cells (CaSki and SiHa) and RNA interference. With the hybridization method, eight microRNAs (miR-9-5p, miR-136-5p, miR-148a-3p, miR-190a-5p, miR-199b-5p, miR-382-5p, miR-597-5p, and miR-655-3p) were found to be expressed differently in the HPV16-positive cervical cancer group and HPV16-positive normal group (fold change ≥ 2). The results of qPCR showed that miR-148a-3p, miR-190a-5p, miR-199b-5p, and miR-655-3p levels significantly decreased in the cancer group compared with the normal group. Upon silencing of HPV16* E5* and* E6/E7*, miR-148a-3p levels increased in both cell lines. Silencing of* E6/E7* in SiHa cells led to the increase in miR-199b-5p and miR-190a-5p levels. Three HPV16 oncoproteins (E5, E6, and E7) downregulate miR-148a-3p, while E6/E7 inhibit miR-199b-5p and miR-190a-5p expression in cervical carcinoma. The three microRNAs, miR-148a-3p, miR-199b-5p, and miR-190a-5p, may be novel diagnostic biomarkers for cervical cancer development in high-risk HPV-positive patients.

## 1. Introduction

The interaction between viral and host factors is important in cervical carcinogenesis because it triggers tumor growth, invasion, and metastasis. Specifically, human papillomavirus (HPV) infection has been shown to be the most important factor in cervical carcinogenesis: the transformation from normal cervical epithelium to cervical cancer tissue is most likely caused by HPVs, which are episomal, double-stranded DNA viruses that induce epithelial lesions. The oncogenic potential of high-risk HPV is mostly attributed to the products of three early genes:* E5*,* E6*, and* E7*. E6 and E7 exert their oncogenic effect by destabilizing and degrading retinoblastoma protein (pRB) and p53 [1–6], while E5 is thought to play a role during the early steps of transformation in the basal layers of the epithelium and enhance the oncogenic effect of E6 and E7 [7, 8].

Almost all cervical cancers are associated with HPV; however, the majority of women infected with this virus do not develop cervical cancer. Therefore, to detect cervical cancer in high-risk HPV-infected patients, tumor markers that reflect the virus-induced cancerous changes are needed. The importance of epigenetic regulatory mechanisms has become evident in the last decade, and the epigenetic dysregulation of oncogenes and tumor suppressors is the focus of active research. MicroRNAs (miRNAs) function as regulators of different cell processes, such as apoptosis, cell cycle progression, metastasis, and chemo- and radio-resistance [9, 10]. However, the interaction between viral factors, such as early oncoproteins, and host factors, such as dysregulated miRNA expression, during cervical carcinogenesis is still poorly understood [11–13].

In this study, we aimed to identify potential miRNAs that may serve as tumor markers for the early detection of cervical cancer in high-risk HPV-positive patients. Additionally, we investigated the association between high-risk HPV oncoproteins and the dysregulation of miRNAs in cervical cancer cells.

## 2. Materials and Methods

### 2.1. Study Samples and Nucleic Acid Extraction

This study comprised 41 cervical tissue samples. We obtained 26 formalin-fixed paraffin-embedded (FFPE) and 15 frozen cervical tissues samples from the Korea Gynecologic Cancer Bank, Yonsei University College of Medicine, Seoul, Republic of Korea. We divided the collected samples into three control groups (HPV-negative normal tissues [NN], HPV-negative cancer tissue [NC], and HPV16-positive normal tissue [PN]) and one experimental group (HPV16-positive cancer tissue [PC]). Detailed information about these samples is reported in Table 1. All cancer samples were squamous cell carcinomas, which comprise about 80% of all cervical cancers. HPV infection was confirmed using the Abbott Real*T*ime High-Risk HPV PCR assay kit (Abbott Molecular, Abbott Park, IL, USA).

Total RNA was extracted from the frozen tissues using the Labozol reagent (CosmoGenetech, Seoul, South Korea) and from the FFPE tissues using the miRNeasy FFPE kit (Qiagen, Valencia, CA, USA). RNA was quantitated using a Nanodrop2000c spectrometer (Thermo Scientific, Wilmington, DE, USA) and DS-11 (DeNovix, Wilmington, DE, USA) spectrophotometers, and the quantitation and quality of the isolated samples were confirmed using the Agilent 2100 Bioanalyzer (Agilent Technologies, Santa Clara, CA, USA).

### 2.2. miRNA Screening Using the Hybridization Method

RNA was extracted from 12 FFPE cervical tissue samples consisting of three samples from each of the NN, PN, NC, and PC groups. Total RNA from each sample (100 ng) was prepared as instructed in the nCounter miRNA Expression Assay (NanoString Technologies, Seattle, WA, USA) user manual. Mature miRNAs were ligated to a species-specific tag sequence (miRtag). After enzymatic purification of nonligated miRtags, the prepared samples were hybridized using the nCounter Human v3 miRNA Expression Assay CodeSet containing 800 human miRNA hybridization probes. After hybridization, the excess probes were removed by two-step magnetic bead-based purification on the nCounter Prep station. Specific target molecules were quantified using the nCounter Digital Analyzer, by counting individual fluorescent barcodes and assessing target molecule levels. For each sample, a scan encompassing 280 fields of view was performed. The data were collected using the nCounter Digital Analyzer after taking images of the immobilized fluorescent reporters in the sample cartridge (NanoString Technologies).

### 2.3. Quantitative Polymerase Chain Reaction (qPCR) Analyses of Clinical Samples

To investigate the changes in miRNA expression levels, we performed qPCR analysis of the samples obtained with the hybridization method. Additionally, we validated eight miRNAs in the 29 clinical tissue samples that had not been used in the miRNA hybridization (4 PN and 25 PC). Reverse transcription was performed using the miScript II RT Kit (Qiagen, Hilden, Germany) according to the manufacturer's instructions. qPCR was performed on the StepOnePlus Real-Time PCR System (Applied Biosystems, Carlsbad, CA, USA) using the 2× QuantiTect SYBR Green PCR Master Mix (Qiagen). Thermal cycling conditions were as follows: 95°C for 15 min, followed by 40 cycles of 94°C for 15 s and 55°C for 30 s, and 70°C for 30 s. The data was analyzed using the StepOne software v2.2.2 (Applied Biosystems). All qPCR reactions were run in triplicate, and gene expression levels of each miRNA were normalized to the levels of the endogenous control small RNA U6, using the 2^-ΔΔCt^ method.

### 2.4. HPV16 E5/E6/E7 Silencing in Cancer Cells In Vitro

We investigated the effect of HPV16 oncoproteins on human miRNA expression using two human cervical cancer cell lines: HPV16-positive CaSki cells (ATCC CRL-1550; American Type Culture Collection [ATCC], Manassas, VA, USA) and SiHa cells (ATCC HTB-35; ATCC). Cervical cancer cells were cultured in RPMI-1640 medium (Gibco BRL, Grand Island, New York, USA), supplemented with 10% fetal bovine serum (Gibco BRL) and 1% penicillin/streptomycin under 5% CO_2_ at 37°C. To investigate the role of HPV16 E5, E6, and E7 on the expression of miRNAs during cervical carcinogenesis, we silenced the* E5* gene using small hairpin RNA (shRNA) overexpressed by lentiviral vectors and the bicistronic* E6/E7* genes using small interfering RNA (siRNA) in cervical cancer cells, as previously reported [14, 15]. Scrambled shRNA or siRNA sequences were used as a negative control.

Cells were transfected/infected in 12-well plates and collected 0, 24, 48, and 72 h after. Total RNA was extracted with the TRIzol reagent (Invitrogen, Carlsbad, CA, USA). The efficiency of knockdown was determined by measuring the expression levels of HPV16* E5*/*E6*/*E7* mRNA three times by qPCR at 72 h after transfection/infection. The levels of miRNA expression were also determined using qPCR at the indicated time-points. Primer sets described previously [14] were used to amplify each miRNA. Glyceraldehyde 3-phosphate dehydrogenase (*GAPDH*) was used as an internal control.

### 2.5. Data Analysis

For miRNA profiling, the reporter counts were collected using the nSolver software v3.0.22 (NanoString Technologies). miRNA profiling data were normalized by positive reaction controls to a panel of five housekeeping genes (actin B [*ACTB*], *β*2 microglobulin [*B2M*],* GAPDH*, and ribosomal proteins [*RP*]* L19* and L10) and to miRNA-23a and miRNA-191 [16]. The R software v.3.1.1 was used for analysis and graphics construction [17]. Differences between the samples were considered significant at a fold change ≥ 2 and* P* ≤ 0.01. For qPCR analysis, all data were expressed as the mean ± standard deviation. Statistical differences between the groups were assessed using Student's two-tailed* t*-test.* P*< 0.05 was considered to indicate a statistically significant difference.

## 3. Results

### 3.1. miRNA Profiling in Cervical Tissues

**Figure 1:**
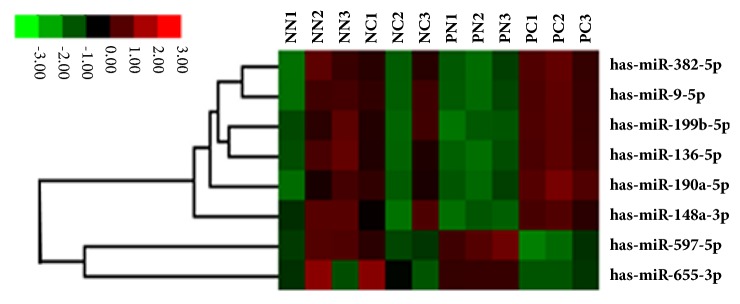
**Differential miRNA expression in cervical tissues.** Heat map indicating the eight differentially expressed miRNAs in cervical cancer tissues. NN, HPV16-negative normal; NC, HPV16-negative carcinoma; PN, HPV16-positive normal; PC, HPV16-positive carcinoma.

The expression profile of 800 human miRNAs in the PC and PN groups was analyzed using the hybridization method. We identified 99 differentially expressed miRNAs (fold change ≥ 2 and* P* ≤ 0.01) between the two groups. Among these, eight miRNAs had significantly different expression in the PC group compared with pooled control group (combined NN, NC, and PN groups). Six miRNAs were upregulated and two were downregulated in the PC group compared to the control group.  Figure 1 shows the heat map indicating these eight differentially expressed miRNAs.

### 3.2. Differentially Expressed miRNAs in Cervical Cancer

**Figure 2:**
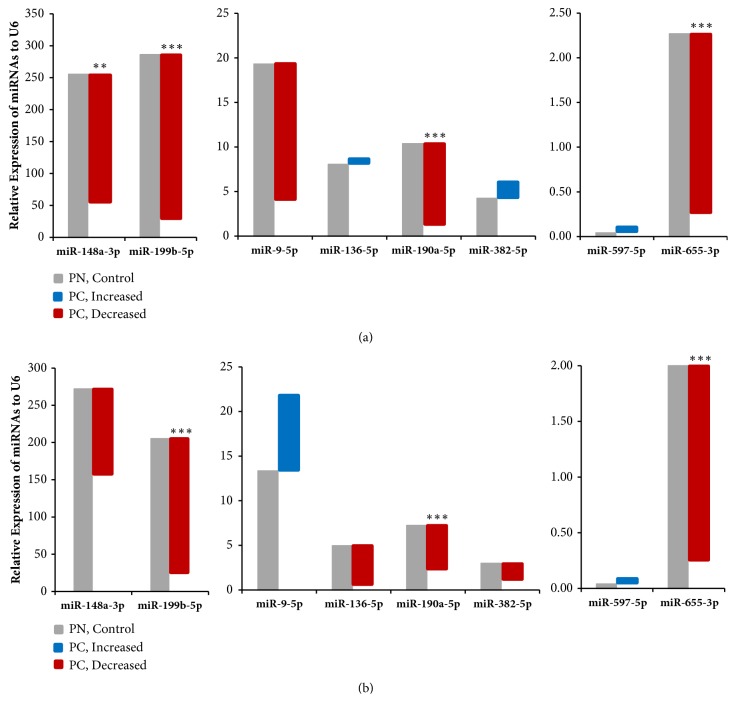
**Relative expression of selected miRNAs in cervical tissues.** (a) Expression of the indicated miRNA assessed with the hybridization method (3 PN vs. 3 PC). (b) Expression of the indicated miRNA assessed with the hybridization method in additional samples (4 PN vs. 25 PC). PN, HPV16-positive normal; PC, HPV16-positive carcinoma. *∗*,* P*< 0.05; *∗ ***∗**,* P*< 0.001; *∗∗∗*,* P*< 0.0001.

To verify the results of the miRNA expression profiles, we reevaluated the expression of the eight identified miRNAs, by qPCR. We found that miR-148a-3p, miR-190a-5p, miR-199b-5p, and miR-655-3p levels were significantly decreased in the PC group compared to the PN group (respective 0.22-fold, 0.11-fold, 0.11-fold, and 0.11-fold), while the levels of other miRNAs did not significantly differ between the two groups (Figure 2(a)). Notably, only the results regarding miR-655-3p expression were consistent with those obtained using the hybridization method. Analysis of additional clinical tissue samples showed that miR-190a-5p, miR-199b-5p, and miR-655-3p expression was significantly decreased in the PC group compared with the PN (control) group (respective 0.32-fold, 0.12-fold, and 0.18-fold; Figure 2(b)).

**Figure 3:**
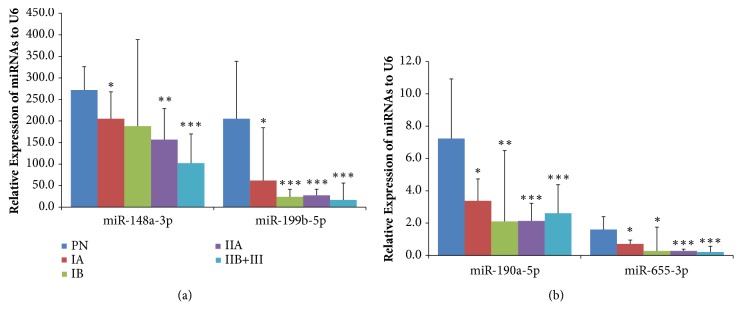
**Relative expression of selected miRNA according to the International Federation of Gynecology and Obstetrics (FIGO) stages of the samples.** The HPV16-positive cancer (PC) group (IA [n = 4], 1B [n = 12], IIA [n = 5], IIB+III [n = 6]) were compared with the HPV16-positive normal group (PN; n = 5). *∗*,* P *< 0.05; *∗∗*,* P *< 0.001; *∗∗∗*,* P *< 0.0001.

Four miRNAs, whose expression was significantly different in the PC and PN groups, were analyzed according to the International Federation of Gynecology and Obstetrics (FIGO) staging system classification of the tissue samples (Figure 3 & Table 2). We found that the expression of these miRNAs in the PC group significantly decreased in almost all FIGO stages compared to the PN group, with the exception of miR-148a-3p expression in the IB group, characterized by clinically visible lesions confined to the cervix (stromal invasion > 5.0 mm in depth or >7.0 mm in horizontal spread). The relative expression folds for each FIGO stage compared to the PN group are shown in detail in Table 2. Furthermore, the expression levels of these miRNA were shown to decrease gradually as the disease progressed.

### 3.3. HPV16 E5/E6/E7 Effect on Host miRNA Expression

**Figure 4:**
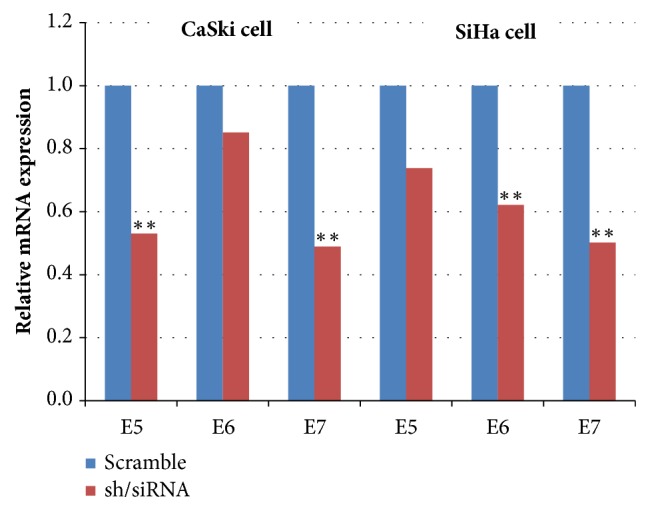
***E5*,* E6,* and* E7 *expression levels after RNA interference. **Relative expression of* E5*,* E6*, and* E7 *mRNA 72 h after shRNA or siRNA-mediated silencing in CaSki and SiHa cells.* GAPDH *was used for normalization. *∗*,* P *< 0.05; *∗∗*,* P *< 0.01.

**Figure 5:**
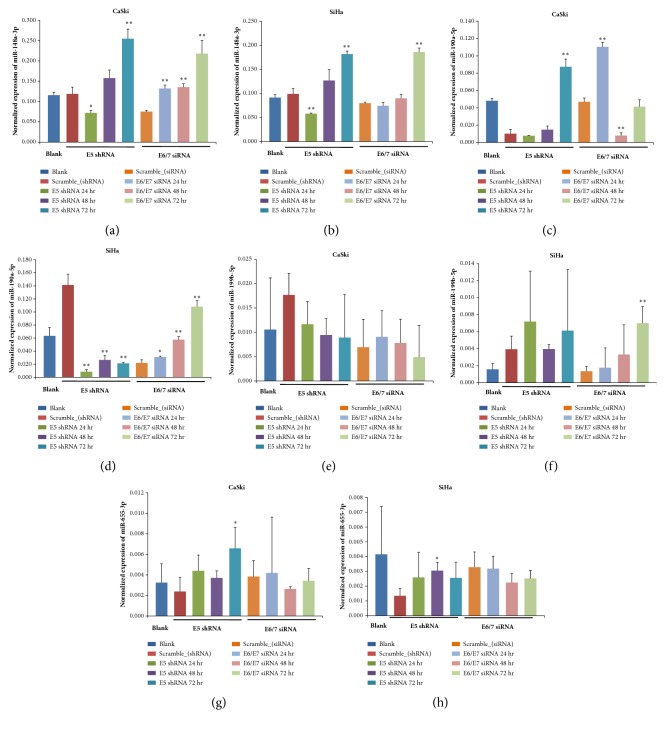
**Effects of* E5* and* E6/E7* silencing on the expression of selected miRNAs. **The expression of the indicated miRNA was assessed by qPCR in CaSki and SiHa cells at different time points after* E5* and* E6/E7* silencing.* GAPDH* was used for normalization. *∗*,* P *< 0.05 and *∗∗*,* P *< 0.01, compared with the scramble control samples.

HPV16* E5*,* E6,* and* E7* silencing reduced the expression of these genes by 0.53-fold, 0.85-fold, and 0.49-fold in CaSki cells and by 0.74-fold, 0.62-fold, and 0.50-fold in SiHa cells, compared to control samples, overexpressing scramble shRNA or siRNA (Figure 4). We then determined the relative expression levels of the selected four miRNAs in Ca Ski and SiHa cervical cancer cells and compared them with the miRNA levels in control samples, at the indicated time points after transfection/infection. miR-148a-3p expression significantly increased in both cell lines 72 h after gene silencing, especially in the* E6/E7* knockdown group, while miR-199b-5p showed a variable expression pattern and its levels were significantly increased only in SiHa cells 72 h after* E6*/*E7* silencing. miR-190a-5p expression significantly increased in CaSki cells 72 h after* E5* silencing and in SiHa cells72 h after* E6*/*E7* silencing. However, no significant changes in miR-655-3p levels were observed (Figure 5).

## 4. Discussion

The correlation between changes in miRNA expression and cervical cancer development was originally described in 2009 [18]. The authors of the study showed that the expression of miR-21 promotes HeLa cell proliferation, while its inhibition suppresses cell proliferation by inducing the overexpression of the tumor-suppressor gene programmed cell death 4 (PDCD4), a programmed cell death protein. miR-21 was subsequently demonstrated to be an important oncomir, overexpressed in a wide variety of cancers, including cervical cancer [19].

Several miRNAs, such as miR-34a, miR-886-5p, miR-143, miR-203, and miR-155, have been shown to have differential expression in cervical cancer and normal samples [20–24]. Therefore, miRNAs have been studied as potential diagnostic biomarkers in cancer development and progression and as therapeutic targets for cervical cancer treatment [25–27]. In 2014, Sharma* et al*. [28] reviewed 246 differentially expressed miRNAs involved in cervical cancer progression.

However, to date, no miRNAs are used practically as markers for the diagnosis of cervical cancer. One of the possible reasons is the lack of consistency in the research data, which makes it difficult to determine the clinical value of the identified miRNA. Inconsistency in miRNA expression levels in cervical carcinogenesis may be attributed to patient-intrinsic variation, time and temperature changes during sample collection, processing, contamination by cells and blood components, RNA extraction method used, normalization, and storage time and conditions [29]. Another important issue is the necessity to optimize the technology to apply microRNA gene expression analysis to clinical practice; specifically, for high sensitivity, high specificity, and technical reproducibility, low cost and proper outputs are required.

In this study, we focused on the most prevalent HPV type (HPV16) and cervical squamous cell carcinoma (FIGO stages IB1 ~ III). We screened 800 human miRNAs, to identify potential novel biomarkers and investigated the effects of HPV16 oncoproteins on selected miRNAs. We analyzed the results obtained in HPV16-positive cancer samples and compared them to those from HPV16-positive healthy individuals, to identify early diagnostic markers for cancer development in high-risk HPV-infected patients. Our study has several limitations, such as the difficulty of relying on the results obtained using banked tissue samples, due to the inability of controlling preanalytical factors. Different conditions associated with sample processing, storage, RNA extraction, and collection time, all important determinants of miRNA stability, were used to collect the samples, from May 2012 to March 2017. Additionally, the samples initially tested for screening included high-grade squamous intraepithelial lesions, a noninvasive cancer.

We identified eight miRNAs as putative biomarkers in HPV16-positive cervical cancer tissues: miR-9-5p, miR-136-5p, miR-148a-3p, miR-190a-5p, miR-199b-5p, and miR-382-5p (upregulated) and miR-597-5p and miR-655-3p (downregulated). It has previously been reported that miR-148a expression is altered during cancer development and may serve as a specific marker for HPV-induced malignancy [30, 31]. miR-136 has been shown to be downregulated in minimal deviation adenocarcinomas of the uterine cervix, while miR-9 is upregulated in cervical cancers, and its upregulation is associated with lymph node metastases and vascular invasion [32, 33]. miR-199b-5p has been reported to be downregulated in squamous cell carcinoma and is associated with poor prognosis [34].

Among the eight identified miRNAs, miR-148a-3p, miR-190a-5p, miR-199b-5p, and miR-655 expression was shown to be significantly suppressed in the PC group by qPCR. The results for miR-148a, miR-190a, and miR-199b obtained with the hybridization and qPCR methods were discordant. One possible reason for the discrepancy is that the reference genes used to normalize the data were different in the two methods. All reference genes were selected according to previously published studies [14, 16]. Notably, the difference in the expression levels might depend on the instability of specific miRNAs. Specifically, the stability of nucleic acids extracted from FFPE samples may be reduced. While we verified the quality of extracted RNA, it should be noted that we evaluated the quality of total isolated RNA, not that of specific miRNAs. Finally, the small number of samples used for screening increased the chances for nonsignificant results.

On this note, Mestdag* et al*. [35] have compared 12 available platforms for miRNA expression analysis and found that the concordance of miRNA expression was less than 70% between hybridization and qPCR. Particularly, the average validation rate of miRNA levels when using any platform combination was only 54.6% (95% confidential interval, 52.5–56.7%). Notably, the silencing efficiency was about 50–60% for* E5* and* E7* in CaSki cells and* E6* and* E7* in SiHa cells. This was due to the difference in HPV16 copy number per cell and because of cell characteristics (such as race and histologic type). The silencing of HPV16* E5* and* E6/E7* was shown to inhibit miR-148a-3p expression in both the cell lines, while the silencing of HPV16* E6/E7* in SiHa cells increased miR-199b-5p and miR-190a-5p expression levels.

## 5. Conclusion

In this study, we found that three HPV16 oncoproteins were associated with the downregulation of miR-148a-3p expression, while HPV16 E6/E7 led to the downregulation of miR-199b-5p and miR-190a-5p in cervical carcinoma. Our results suggest that miR-148a, miR-199b, and miR-190a may be novel biomarkers for cervical carcinogenesis after HPV16 infection.

## Figures and Tables

**Table 1:** Characteristics of the clinical cervical tissue samples.

**Related experiment**	**Group**	**Sample type**	**No. Sample**	**HPV genotype** **∗**	**Bethesda system & FIGO stage (N)**
**Hybridization**	HPV16-negative normal (NN) cervix	FFPE	3	Not detected	-
	HPV16-negative cervical carcinoma (NC)	FFPE	3	Not detected	CIN II (1), CIN III (2)

**Hybridization & 1st qPCR**	HPV16-positive normal (PN) cervix	FFPE	1	16	-
			2	16 & other	-
	HPV16-positive cervical carcinoma (PC)	FFPE	3	16	CIN III (1), IA1 (2)

**2nd qPCR**	HPV16-positive normal (PN) cervix	FFPE	2	16	-
			2	16 & other	-
	HPV16-positive cervical carcinoma (PC)	FFPE	8	16	IA1 (1), 1B1 (1), IB2 (1), IIA (1), IIB (4)
			2	16 & other	IA1 (1), 1B1 (1)
		Frozen	9	16	IB2 (5), IIA (2), IIB (1), III (1)
			3	16 & 18	1B1 (1), IB2 (1), IIA (1)
			3	16 & other	IB2 (2), IIA (1)

*∗*HPV genotyping resulted in four categories: not detected, only HPV16 detected (16), co-infected with HPV16 and HPV18 (16 & 18), and co-infected with HPV16 and high-risk viruses other than HPV16 and HPV18 (16 & other).

Abbreviations: CIN, cervical intraepithelial neoplasia; FFPE, formalin-fixed paraffin-embedded; FIGO, international federation of gynecology and obstetrics; HPV, human papilloma virus; qPCR, quantitative PCR.

**Table 2:** The relative expression fold of selected miRNAs with different FIGO stage.

FIGO stage	miR-148a-3p		miR-199b-5p		miR-190a-5p		miR-655-3p	
(I) Compared with U6	2^-ΔC*т*^	*P* value	2^-ΔC*т*^	*P* value	2^-ΔC*т*^	*P* value	2^-ΔC*т*^	*P* value
PN (control)	282.20		225.89		7.50		1.68	
IA	205.27	0.0385	62.03	0.0125	3.55	0.0075	0.51	0.0053
IB	188.13	0.5729	23.88	<0.0001	2.10	0.0006	0.28	0.0157
IIA	156.64	0.0003	27.69	<0.0001	2.13	<0.0001	0.28	<0.0001
IIB and III	102.17	<0.0001	16.59	<0.0001	2.61	<0.0001	0.21	<0.0001

(II) Compared with control	2^-ΔΔC*т*^	*P* value	2^-ΔΔC*т*^	*P* value	2^-ΔΔC*т*^	*P* value	2^-ΔΔC*т*^	*P* value

IA	0.73	0.0009	0.27	0.0001	0.47	0.0001	0.30	<0.0001
IB	0.67	0.4128	0.11	0.0001	0.28	<0.0001	0.17	0.0056
IIA	0.56	<0.0001	0.12	0.0001	0.28	<0.0001	0.17	<0.0001
IIB and III	0.36	<0.0001	0.07	<0.0001	0.35	<0.0001	0.12	<0.0001

ΔC*т* = Target Cт – RNU6 Cт, 2^-ΔC*т*^ = Normalized target gene amount relative to RUN6 gene amount of each group (multiplied by 1000).

ΔΔC*т* = Target sample ΔC*т* - control sample ΔC*т*, 2^-ΔΔC*т*^ = Normalized gene amount of target group relative to target gene amount of control group.

Abbreviations: FIGO, international federation of gynecology and obstetrics; PN, HPV16-positive normal.

## Data Availability

The [hybridization and qPCR] data used to support the findings of this study are included within the supplementary information files.

## Supplementary Materials

Supplementary Materials Table S1: Raw data_Human microRNA lists (800) and expression levels. Table S2: Raw data_qRT PCR for 8 selected human microRNAs. Table S3: Raw data_Human microRNA expression levels depend on stages. Table S4: Raw data_Relative expression of E5, E6, and E7 mRNA. Table S5: Raw data_E5, E6, E7 silencing effects on human microRNAs.
